# Queer Babies

**Published:** 1889-02-09

**Authors:** 


### Babies.

*(Concluded from p. 281.)*

### VI.—QUEER BABIES.

There can hardly be a doubt that at least in ninety-nine cases out of a hundred the marvellous rigmaroles about the effect of maternal mental impressions on unborn human life have their origin in mere coincidence, crudity of thought, and loose superficial observation. This at least is certainly the opinion of all the best authorities, and every medical man of any experience could tell of cases in which the strongest impressions and the most distressing apprehensions have proved to be quite without apparent effect. Indeed, so common is this experience, and so rare are instances in which there is even in appearance any good reason for supposing that physical peculiarities are due to mental disturbance, that many medical men altogether reject the idea that any amount of thought or reflection, or mental shock in the mother, can develop abnormalities in the child.

On the other hand, however, there are good authorities who are not able to go so far. They agree that cases of modification by mental disturbance are extremely rare, and can occur only in very early stages of development, if at all. But some of them seem bound to admit that there are a few well-authenticated facts which are quite inexplicable on any theory except that of the mental perturbation of the mother having affected the unborn child. Among the most curious cases on record was that of a girl offered for sale in Hatton Garden about the middle of the last century. Her parents were Africans, and were kidnapped in their own country, about 300 miles inland from the Gold Coast. They were quite black, and until they were brought down to the coast for shipment to Virginia, they had never seen a white man. It is easy to believe that when they did see one the sight would be decidedly sensational. They were carried to America, and a short time after landing the woman gave birth to a child, which was a negro in all respects except in the matter of colour. It had the features of the negro and the woolly hair, but the skin colour was that of a European, and the hair was almost as white as snow. The child lived to grow up; and an advertisement in the *Daily Advertiser* of April, 1754, says that any person desirous of being a purchaser may hear of the particulars by applying at a certain number in Hatton Garden. "None will be admitted but those that intend really to purchase; and to prevent unnecessary applications, her price will be four hundred guineas." Of course the wisdom of the day ascribed the phenomenon to the fright of the mother, and if it be assumed that such effects are possible, it is difficult to conceive any experience much more likely to affect offspring than that of this poor negro woman.

As to the spotted children that have been shown from time to time as great curiosities, remembering the rather scurvy trick that Jacob played upon his father-in-law, as described in the thirteenth chapter of Genesis, some may be inclined to think that they are the result of mental impressions. There was a very remarkable instance of this odd freak of nature included in the famous collection of Richardson, the showman. This was a child born in the island of St. Vincent in 1808. His parents also were natives of Africa, and both black. But George Alexander Gratton, as Richardson had him christened, was born with his negro skin and hair spotted all over with brown and white. He was brought to this country and exhibited till he was nearly five years old, when he died. The famous showman is said to have taken an affectionate interest in the child, and when the little wanderer died, had a monument set up to his memory in Great Marlow, in Buckinghamshire, where he was buried. A similar phenomenon was shown in this country in the person of "the piebald boy Primrose"; and we believe such oddities of skin colour are not altogether infrequent when blacks and whites are in association. The mulatto is, of course, the ordinary result of a mingling of races, but now and again the commingling of blood, instead of modifying the pigment cells of the skin generally, affects it only in patches.

In order to bring about modifications of colour, either as in the mulatto or the spotted boy, it is, of course, not necessary that the actual parents shall be of different races. It may have been the grandparents, or even further back. In the human world Nature will often do as she does in the vegetable world—hark back to previous types, and it is just possible that if the whole truth were known, that white negress we have just been talking about would be much more satisfactorily explained than by attributing her colour to mental shock. White men make their way into a good many odd corners of Africa, and it is said they do not always take a copy of the Decalogue with them. Whether niggers have souls used to be a moot point with many travellers; that they often have very comely bodies is beyond any doubt; and we once heard Livingston affirm that many Europeans were known in the heart of Africa for their looseness and profligacy.

Abnormalities of form are no doubt very generally due to arrested development. Mr. Frank Buckland once made some interesting experiments on the ova of salmon. He found that by dropping a minute particle of dust on the head of the unhatched salmon he could check the natural development, and produce a fish with a malformed head. Similarly, a particle put upon the tail would arrest the development of that part of the creature, and a fish would be hatched with a body and tail out of all proportions with each other; or he could thus bring about the production of a fish without a tail at all. By tampering with the ova he could even hatch out double fishes—two fishes joined together after the manner of the Two-headed Nightingale. That particle of dust pressing on the particular point of the embryo and impeding its nutrition, no doubt illustrates what often happens in higher circles of life, and the physical interference that resulted in two-headed fishes probably has its counterpart in the human system, entirely apart from any influence of mental perturbation. Some of the results of arrested nutrition have been very extraordinary, but they are not pleasant subjects to dwell upon, though some have had almost all the painfulness of their nature neutralised by the marvellous illustrations of human adaptability they have afterwards presented, displaying what human nature is capable of even under the strangest conditions and the direst disadvantages. Little Miss Biffin, who was publicly exhibited in this country in the beginning of the present century, was born without arms or legs; but she learned to do what might have been thought the most impracticable things by her mouth alone. She would cut out and make any part of her own clothes. She could thread her own needle, and sew very neatly; and she could write well. A letter written by her is preserved in the British Museum, and the other day the writer accidentally came across it. She is said to have been able to paint landscapes, and seems to have partly maintained herself by painting miniature portraits on ivory for three guineas each. When the dismayed parents first looked on this queer little stranger, how little could they have imagined that in accomplishments she would one day far surpass many a fine lady with rings on her fingers and bells on her toes. Miss Honeywell was another such an infant, born in New York, and exhibited over here in Oxford Street. She also learned to work with her lips, could do embroidery and net very fine silk purses, and do any kind of fine needlework.

It would be easy to extend the list of abnormalities quite indefinitely. There is hardly a vagary to which the imagination can abandon itself which has not, at some time or other, had its embodiment in an actual freak of Nature. Even the old mythological Cyclops, the being with one eye in the middle of the forehead, has appeared again and again indeed, half the medical museums of London have preserved specimens of this particular malformation, and they present innumerable other irregularities almost as striking. But though such subjects are undoubtedly interesting, they are somewhat painful and unpleasant subjects for description, and perhaps enough has been said about them.

